# Quantifying Chaperone-Mediated Transitions in the Proteostasis Network of *E. coli*

**DOI:** 10.1371/journal.pcbi.1003324

**Published:** 2013-11-14

**Authors:** Alex Dickson, Charles L. Brooks

**Affiliations:** 1 Department of Chemistry, The University of Michigan, Ann Arbor, Michigan, United States of America; 2 Department of Chemistry and Biophysics Program, The University of Michigan, Ann Arbor, Michigan, United States of America; Harvard University, United States of America

## Abstract

For cells to function, the concentrations of all proteins in the cell must be maintained at the proper levels (proteostasis). This task – complicated by cellular stresses, protein misfolding, aggregation, and degradation – is performed by a collection of chaperones that alter the configurational landscape of a given client protein through the formation of protein-chaperone complexes. The set of all such complexes and the transitions between them form the proteostasis network. Recently, a computational model was introduced (FoldEco) that synthesizes experimental data into a system-wide description of the proteostasis network of *E. coli*. This model describes the concentrations over time of all the species in the system, which include different conformations of the client protein, as well as protein-chaperone complexes. We apply to this model a recently developed analysis tool to calculate mediation probabilities in complex networks. This allows us to determine the probability that a given chaperone system is used to mediate transitions between client protein conformations, such as folding, or the correction of misfolded conformations. We determine how these probabilities change both across different proteins, as well as with system parameters, such as the synthesis rate, and in each case reveal in detail which factors control the usage of one chaperone system over another. We find that the different chaperone systems do not operate orthogonally and can compensate for each other when one system is disabled or overworked, and that this can complicate the analysis of “knockout” experiments, where the concentration of native protein is compared both with and without the presence of a given chaperone system. This study also gives a general recipe for conducting a transition-path–based analysis on a network of coupled chemical reactions, which can be useful in other types of networks as well.

## Author Summary

To maintain proper amounts of folded, functional proteins, cells use systems of chaperones to correct misfolded proteins, disassemble aggregates, and provide sheltered environments in which proteins fold to their native structure. Typically, an individual system is studied in isolation, and its effects on a given protein are studied using “knockouts”, where the amount of native protein is compared with and without the active chaperone system. However, when multiple chaperone systems are operating simultaneously, knockouts can fail to reveal chaperone activity, as different chaperone systems can compensate for one another. We use a previously introduced computational model of chaperone systems in *Escherichia coli*, in combination with our transition-path analysis methods for networks, to analyze paths of individual proteins through the set of possible chaperone-bound and -unbound states. Our analysis allows us to answer questions that are inaccessible to knockout experiments, such as: How often will a given chaperone system be used to rescue a protein from a misfolded state? This approach provides a clear view of how the different systems of chaperones cooperate and compete under varying conditions.

## Introduction

Protein homeostasis (proteostasis) is essential for the viability of an organism. The disruption of protein homeostasis involving the misfolding and subsequent aggregation of proteins is implicated in many diseases, including Down's syndrome, type-II diabetes, Alzheimer's, Parkinson's and Huntington's disease [1]–[4]. In addition, inherited mutations that lead to excessive degradation of proteins can lead to loss-of-function diseases, such as cystic fibrosis and Gaucher disease [2], [5]. Thus, in every living cell, a system of chaperones – called the proteostasis network – has evolved to help proteins fold, correct or clear misfolded protein, and prevent (or even reverse) the formation of protein aggregates.

Proteostasis networks can be broken down into chaperone subsystems (such as the Hsp60, Hsp70 and Hsp90 systems in eukaryotes) [4], and these systems can be studied individually. Much work has focused on cataloguing the proteins that are clients of these different chaperone systems, and examining their structural features [6]–[9]. The molecular mechanisms of interaction of chaperones with client proteins in each system has been studied [10]–[15]. Data from experiment has been synthesized into theoretical models, which describe the passage of client proteins through a given chaperone system [16]–[18].

However, different chaperone systems do not operate in isolation *in vivo*. Most chaperone activity relies on the consumption of ATP, which is derived from a shared source. Also, chaperones can have more than one function; DnaK in prokaryotes can bind both to unfolded or misfolded protein in order to prevent aggregation, or it can help prepare aggregates for binding to another chaperone, ClpB, for disaggregation. Complicating matters is that proteins are not selective in the chaperone system to which they bind: it has been shown that there is significant overlap in the sets of client proteins whose solubilities increase under the action of different chaperone groups [19]. Furthermore, experimental studies have shown the synergistic action of multiple chaperone groups [9], [19], [20]. Thus, chaperones form complex networks of interaction in the cell. This motivates a holistic, systems-based approach, in which experimental data from a variety of contexts is synthesized to study the proteostasis network in its entirety.

This is precisely the goal of FoldEco [21]: a recently presented tool that describes the proteostasis network in *Escherichia coli*. FoldEco synthesizes previously established models of various chaperone systems into a single network of reactions, whose rates are parameterized using experimental data. Dynamics on the FoldEco network describe the synthesis, folding, unfolding, misfolding, aggregation and degradation of a client protein, as well as the passage of the client protein through three chaperone systems, which work to correct misfolded structures, prevent aggregation and maintain a population of functional native protein. The FoldEco program uses a set of initial conditions and reaction rates to propagate the concentrations of the different species in the network forward in time. However, lost in this approach is the ability to track the trajectories of single molecules, which would allow us to answer fundamental questions such as: how often is a given chaperone system used to get from one point in the network to another?

We have developed a network analysis technique that quantitatively determines mediation probabilities in complex networks (i.e., how often a state A is found on transition paths from B to C). This analysis was previously used to detect hub-like activity in protein configuration space networks, and was referred to as “hub scores” [22], [23]. Here, we show how mediation probabilities can be calculated from the output of the FoldEco program by constructing transition matrices for states of the client protein. We show that these probabilities can provide insight into proteostasis networks by revealing how often different competing pathways, involving different chaperone systems, are used to connect different regions of the network, such as the misfolded and unfolded states of the client protein. For client proteins, we choose four characteristic biophysical protein profiles based on the Monte Carlo results of Powers et al [21], which demonstrate a range of characteristic behaviors. We calculate the relative probabilities of taking transition paths through each chaperone system for the four different protein types, and demonstrate how these probabilities change as a function of system parameters, such as the protein synthesis rate, and the total chaperone concentration.

## Results

### Four characteristic protein profiles

In the FoldEco model, there are a large number of parameters that can be adjusted in order to more accurately describe the activity of a particular protein. As exploring this entire parameter space is infeasible, in Powers et al. [21] a subset of six variables were chosen, and the resulting six-dimensional space was explored using a large number of points chosen by Monte Carlo sampling. The six variables comprise folding rate constants (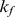 and 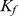), misfolding rate constants (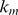 and 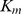), and well as two parameters that control the aggregation propensity of the protein (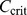 and 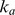). We instead study a small number (four) of proteins in depth, which are chosen to demonstrate a range of preferences for the different chaperone systems. Using the results of the Monte Carlo study, Powers et al. determined biophysical profiles of optimal substrate proteins for the GroELS system while the KJE system is present, and vice versa. They were chosen based on the percent increase of native concentration upon the addition of either the GroELS or the KJE chaperone system (in the presence of the other). We choose parameters from the distributions of the optimal GroELS and KJE substrates to define two of our client proteins. As the optimal GroELS substrates characteristically showed slow folding rates, we refer to the GroELS protein as “Slow Folder”. Similarly, the optimal KJE substrates characteristically show high misfolding rates, and we refer to the KJE protein as “Bad Folder”.

We also define a protein using the average biophysical profile of a set of proteins that were found to aggregate at a low synthesis rate (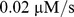) [21]; we refer to this protein as “Aggregator”. Finally, a fourth protein is defined using values of the six parameters that are intermediate between the three proteins defined so far. Since these values are close to the default values given by the FoldEco program, we refer to this protein as “Default”. The values of these 6 parameters are given for each of the four proteins in Table 1.

**Table 1:** Biophysical profiles of the four characteristic proteins.

	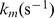	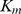	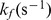	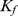	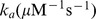	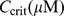
Default	1	100	0.1	10000	0.1	0.1
Slow Folder	1	0.1	0.02	300000	0.4	1
Bad Folder	1	200	0.1	20000	1	2
Aggregator	10	40	0.1	20000	10	0.01

Values are chosen from distributions of optimal substrates given in Powers et al. [21], as described in the main text. 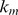 and 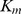 are the rate and equilibrium constant for misfolding, respectively. Larger values of 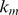 indicate faster misfolding (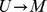), and larger values of 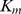 indicate more stable misfolded states. 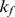 and 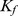 are the rate and equilibrium constant for folding, respectively. Larger values of 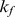 indicate faster folding (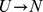), and larger 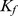 values indicate more stable native states. 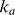 is the aggregation rate, and 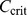 is the critical concentration for aggregation, which controls the disaggregation rate as 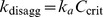. Larger values of 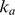 indicate faster aggregation, and larger 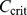 indicates lower stability of the aggregated states.

### Correcting misfolded states

**Figure 1:**
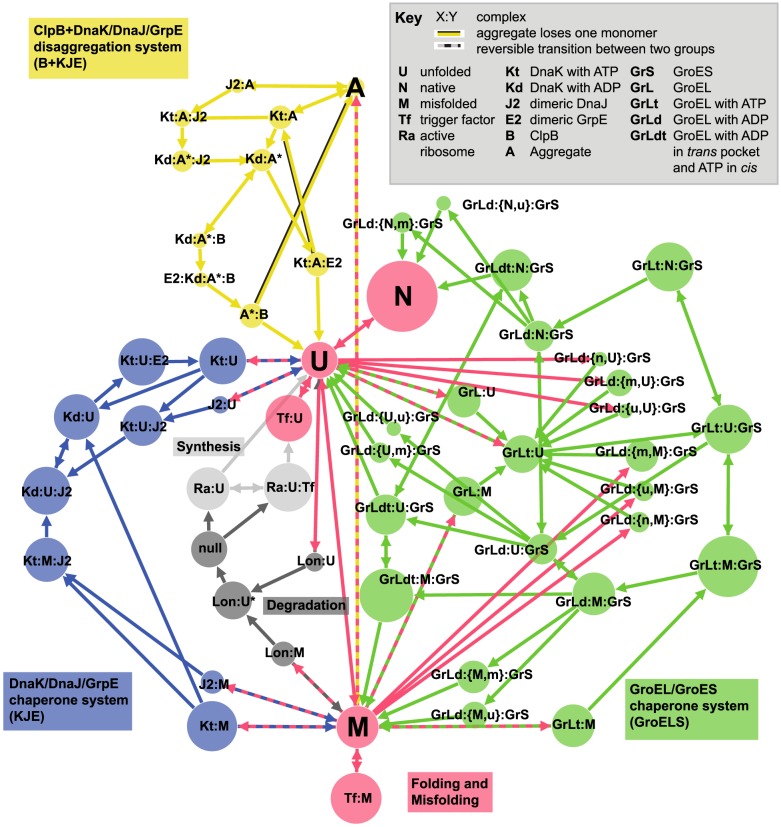
Network of client states in the proteostasis network of *E. coli*. The size of each node is proportional to the logarithm of its concentration after running the FoldEco model with a given set of parameters (the “Default” protein, at 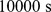, and a synthesis rate of 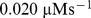, see Section “Four characteristic protein profiles” for more information). The nodes are colored to highlight the different chaperone systems in the network. Colons placed between two, three or four species denote complexes, and the special notation “GrLd:{X,Y}:GrS” denotes a protein in the X state bound in the *cis* ring of the GroEL/GroES complex, and a second protein in the Y state bound in the *trans* ring. As these states are separated into two, depending on whether we are tracking the X, or the Y protein (see Section “Extracting a rate matrix”), we denote the resulting two states as GrLd:{X,y}:GrS and GrLd:{x,Y}:GrS, with the capitol letter marking which protein molecule we are tracking. For simplicity, aggregates of all sizes are denoted here by A, although in practice each aggregate size from 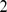 to 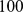 here is given a unique state. There are reversible transitions between aggregates of size 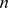 and size 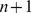, which are not shown here. 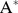 denotes an aggregate that has been prepared for ClpB binding, and 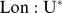 denotes that the protein is now committed to degradation.

Using mediation probabilities, we determine how often certain transition pathways are used between two given states in the FoldEco network. An overview of the network is given in Figure 1, and more information on its construction and the FoldEco model is given in Methods. In this section, we study how the network corrects misfolded states (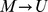 transitions in Figure 1). With all three chaperone systems active, there are five distinct transition paths possible from the misfolded state to the unfolded state. These transitions are: direct, GroELS-mediated, KJE-mediated, B+KJE-mediated, and degradation followed by re-synthesis. We study how often these pathways are visited as a function of synthesis rate. As the synthesis rate increases, correcting misfolded states becomes increasingly important if aggregation is to be prevented. The synthesis rate is controlled through the ribosome activation rate (rate constants 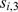 and 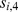 in Powers et al. [21]), which takes on the values of 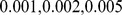 and 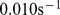, resulting in synthesis rates of 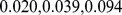 and 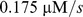, respectively.

We initialize the simulation with no client protein, and a fixed concentration of chaperone species (given in Table 2). The simulation is stopped at 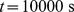 and the concentrations at that time are used to construct the rate matrix used for analysis as described in Methods. We found that after 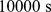, the native protein concentrations have reached equilibrium and the protein dynamics are approximately steady state in systems that do not feature runaway aggregation.

**Table 2:** Chaperone concentrations.

	Conc. ( 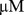 )
Ribosomes	20
Trigger factor	20
DnaK	30
DnaJ	1
GrpE	15
GroEL	42
GroES	35
Lon	0.3
ClpB	1.8

These chaperone concentrations were previously obtained by Powers et al. [21] using geometric averaging of reported experimentally measured values. Note that in Section “GroELS-mediated folding” the concentrations of GroEL and GroES are reduced by multiplying by a factor between 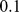 and 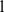.

**Figure 2:**
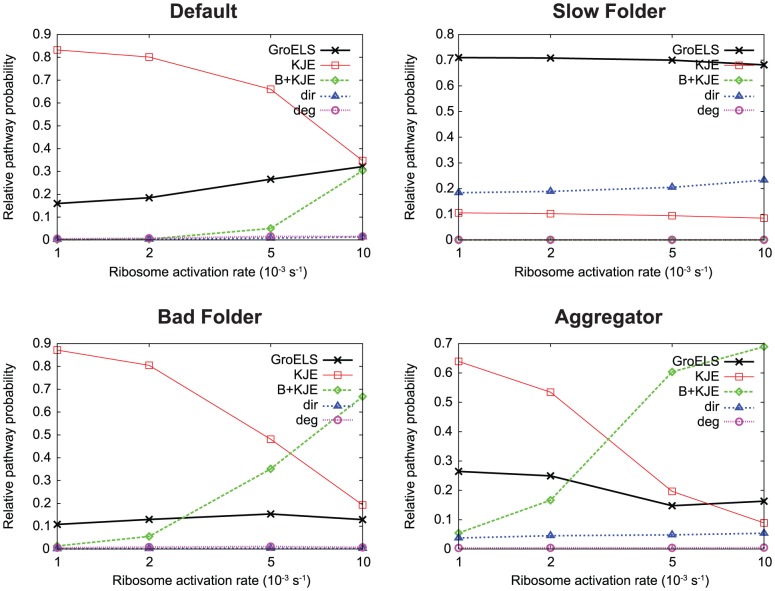
Probability breakdowns of 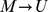 pathways. The relative pathway probabilities are studied as a function of synthesis rate for the four characteristic proteins examined here. “dir” labels the direct 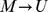 flux, and “deg” labels the 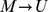 flux occurring by degradation followed by resynthesis. The biophysical profiles for the four proteins are given in Table 1.

**Figure 3:**
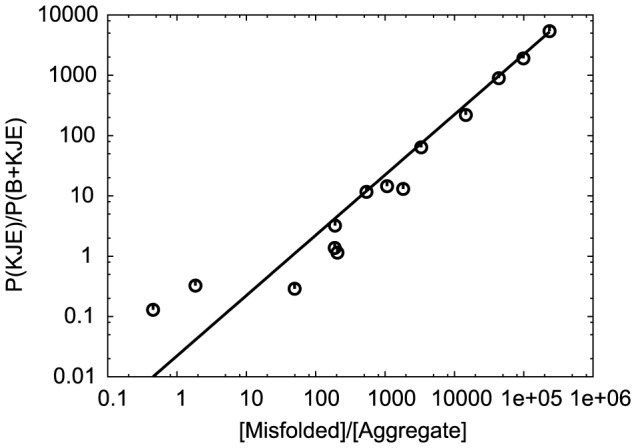
Comparison of the ratios of populations of the KJE and B+KJE pathways from M to U with the concentrations in the misfolded and aggregate states. The points are computed from all proteins at all synthesis rates for which the concentration in the aggregate state (and hence the population of the B+KJE pathway) is greater than zero. The solid line is the best fit to the function 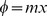, with 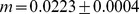, where 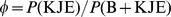, and 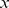 is the ratio of the concentration of misfolded and aggregated protein.

The relative pathway probabilities for the four characteristic proteins are shown in Figure 2. The Default, Bad Folder, and Aggregator proteins show similar behavior: at low synthesis rate, the clearance of misfolded protein is mostly governed by the KJE system, and this responsibility is shifted gradually to the B+KJE system as the synthesis rate increases. This is expected, since the KJE and B+KJE systems produce unfolded protein, via similar mechanisms, from misfolded and aggregated protein, respectively. More aggregated protein is present at higher synthesis rates, which causes the fraction of misfolded protein cleared by B+KJE to increase (Figure S1 shows native, unfolded, misfolded and aggregated protein concentrations). We test this hypothesis by comparing the ratio of activity of the KJE and B+KJE systems with the ratio of the concentrations in the misfolded and aggregated states (Figure 3). The ratio 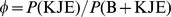 can be fit to the function 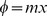, where 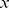 is the ratio of the concentration of misfolded and aggregated protein. This is consistent with the hypothesis that the relative probability of the two pathways is governed by the relative concentrations of their starting points. The proportionality constant, 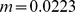, indicates that if the concentration ratio of misfolded to aggregate protein is 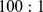, then the KJE pathway (from M to U) is preferred over the B+KJE pathway by a factor of about 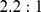.

The crossover from the KJE pathway to the B+KJE pathway is shifted to lower and lower synthesis rates as we move from Default to Bad Folder to Aggregator. This can be explained by the 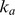 values of the three proteins: 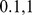 and 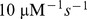, respectively. For a given synthesis rate, the relative population of aggregates grows with larger 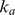, which would lead to increased usage of the B+KJE system, as shown in Figure 3.

**Figure 4:**
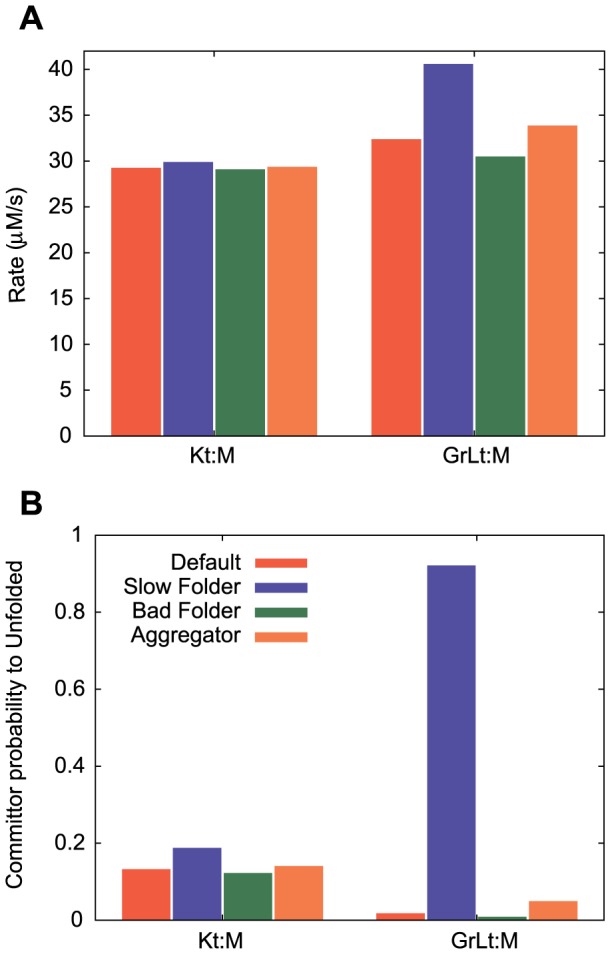
In-depth analysis of M to U transitions. (**a**) Comparison of transition rates from the misfolded state into the 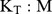 state (in the KJE system) and the 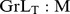 state (in the GroELS system), for all four model proteins. (**b**) Comparison of committor probabilities from the 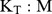 and 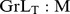 states to the unfolded state. In other words, these are the probabilities that, starting from either 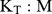 or 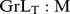, the unfolded state will be reached before the misfolded state.

The Slow Folder protein shows markedly different behavior. The highest probability 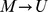 pathway is the GroELS system, and the probabilities are roughly constant as a function of synthesis rate. As the Slow Folder protein was chosen as the ideal client for the GroELS system, this is not surprising, but the question remains as to specifically why the GroELS pathway is favored. The main entries from the misfolded state into the KJE and GroELS systems, respectively, are the 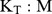 (misfolded protein associated with ATP-bound DnaK) and 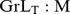 (misfolded protein associated with ATP-bound∶GroEL) states. As such, we compare the entrance rates into these states from the misfolded state at the lowest synthesis rate (Figure 4a). Although the entrance rate into the GroELS cycle is about 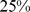 higher for Slow Folder as compared to the others, this is not sufficient to explain the drastic difference in path preference shown in Figure 2. Figure 4b shows committor probabilities starting from the 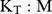 and 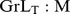 states. These are the probabilities of reaching the unfolded state before the misfolded state given a starting point in either 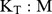 or 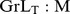, and are computed from the 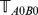 matrices described in the Methods section “Getting mediation probabilities”, where states 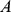 and 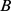 in this context are the misfolded and unfolded states. For Slow Folder, transition paths from 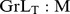 have a much higher likelihood of reaching the unfolded state (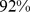) as compared with the other three proteins (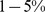). This is due to the fast 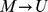 transitions in the GroEL cavity (the rates of which are set to be the same in solution), equal to 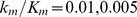 and 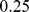 for the Default, Bad Folder and Aggregator proteins respectively, and 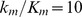 for the Slow Folder protein. Figure 4b also reveals why the KJE pathway is favored for the other proteins. Although the entrance rate into the GroELS cycle is higher than the KJE cycle, the committor probability of reaching the unfolded state is 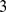 to 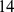 times higher for KJE. This underscores the importance of folding kinetics to the efficiency of the GroELS cycle.

**Figure 5:**
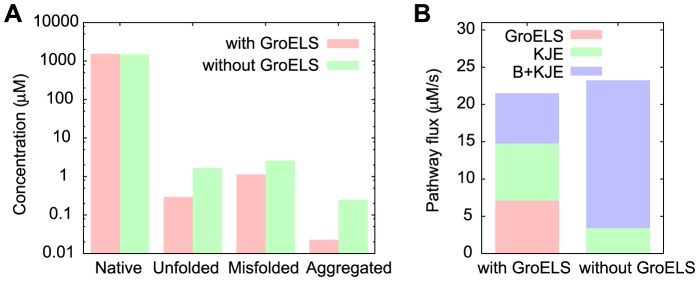
Effects of GroELS knockout on M to U transitions. (**a**) The concentration at 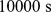 of the native, unfolded, misfolded and total aggregated species both with and without the GroELS system for the Default protein at a ribosome activation rate of 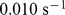. Although the network with GroELS knocked out has higher concentrations of unfolded and misfolded protein, it keeps approximately the same native state concentration. (**b**) Pathway flux from the misfolded to the unfolded state through the three chaperone systems both with and without the GroELS system. We see that the absence of GroELS is more than made up for by enhanced usage of the B+KJE system.

These results give different information than a more conventional “knockout” analysis wherein a particular chaperone system is disabled and the effects on a particular observable is measured – usually either representative of the concentration of native species, or the concentration of aggregated species. To demonstrate this we choose a particular protein and synthesis rate for analysis that is particularly interesting: the Default protein at a ribosome activation rate of 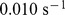. As shown in Figure 2, at this synthesis rate the Default protein uses the chaperone systems GroELS, KJE and B+KJE with approximately equal probability. We then study this protein with the GroELS system knocked out, achieved by setting the initial concentration of GroEL and GroES to zero. The native, unfolded, misfolded and total aggregate concentrations at 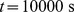 are shown in Figure 5a. We see that the native concentration is approximately unchanged, and the amount of aggregated species is still negligible (the percent of insoluble protein is 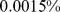 and 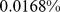 with and without GroELS, respectively), indicating that knocking out the GroELS system would not result in a measurable change in the observables corresponding to the native species or aggregated species concentrations.

Figure 5b shows the contributions to the 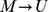 flux by the GroELS, KJE and B+KJE chaperone systems both with and without the GroELS system present. We see that the absence of the GroELS system is more than compensated for by an enhanced contribution of the B+KJE system. Even though the GroELS system is used to clear over 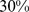 of the misfolded protein under these conditions, its removal had no effect on the native state concentration. This example highlights the advantages of using a transition-path based analysis over knockout experiments when multiple chaperone systems are present. It is also interesting to see that the contribution along the KJE pathway also decreased as GroELS is removed, even though the concentration of its starting state (the misfolded state) increased. This is because the B+KJE pathway uses the chaperones DnaK, DnaJ and GrpE, leaving a lower concentration available for the KJE pathway.

**Figure 6:**
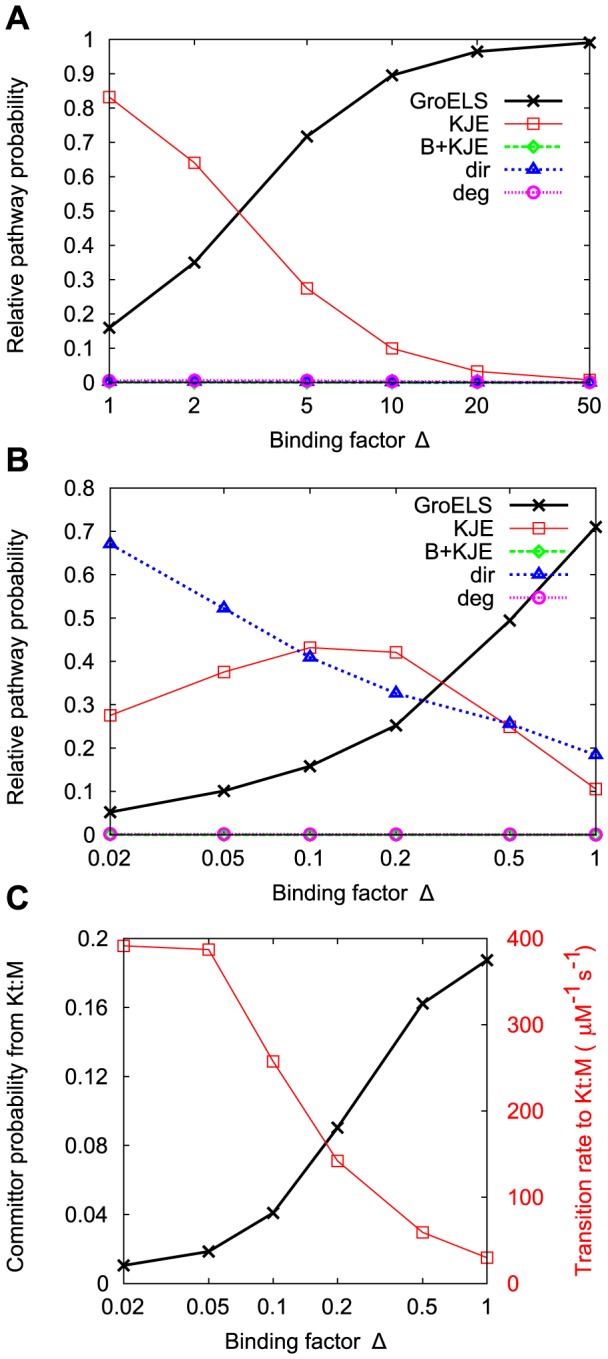
Switching path preferences using binding factors. A multiplicative factor 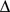 is used to simultaneously modify GroEL and DnaK binding rates for unfolded and misfolded protein. The GroEL-binding rates used here are multiplied by 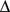, and the DnaK-binding rates are divided by 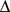. (**a**) The Default protein at low synthesis rate prefers the KJE pathway at 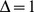, but this preference is shifted to favor the GroELS pathway at high 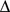 values. (**b**) The Slow Folder protein at low synthesis rate prefers the GroELS pathway at 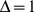, and this preference is only partially shifted to favor the KJE pathway at low 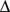 values. (**c**) Although the transition rate into the KJE pathway increases with decreasing 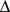, the committor probability (the probability of reaching the unfolded state before returning to the misfolded state) decreases, causing the nonmonotonic behavior in (**b**).

In order to see if binding affinities can govern chaperone preferences, we vary the binding rates to the chaperones DnaK and GroEL and observe the impact on the relative probabilities of each chaperone pathway. The coefficients are varied using a multiplicative factor 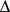. When 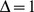, we recover the binding rates used above (which are 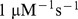 for X to 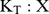, 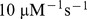 for X to GrL∶X, and 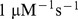 for X to 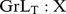, where X is either U or M). The GroEL binding rates are multiplied by 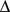, and the DnaK binding rates are divided by 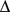, which allows 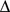 to act as a tuning variable that encourages usage of either the KJE or the GroELS chaperone systems. In Figure 6a, we use the Default protein at the lowest ribosome activation rate (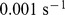), which has a natural preference for the KJE pathway. As 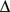 increases, the pathway preference smoothly switches from KJE to GroELS, crossing over between 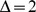 and 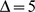. In Figure 6b, we use the Slow Folder protein at the lowest synthesis rate, which has a preference for the GroELS pathway. As 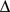 decreases (from right to left), the KJE probability initially increases, and then decreases for 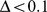. This behavior is counter-intuitive: why would increasing the DnaK binding rate discourage usage of the KJE pathway? Figure 6c demonstrates that even though the binding rate increases with decreasing 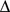, the committor probability from 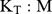 to the unfolded state decreases. This is due to the decreasing concentration of free DnaJ, which is spent by the formation of 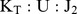 and 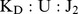 complexes which can result from unproductive binding of unfolded protein to DnaK (Figure S2). Nevertheless, substantial switching of path preferences can again be achieved by adjusting the binding affinities by a factor between 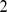 and 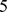. This suggests, for the M to U transition, that relative usage of the two chaperone systems can be controlled through modest adjustments of the relative binding affinities to the two chaperones.

### GroELS-mediated folding

We now study mediation of the 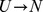 (or folding) transition. This is less complicated than the 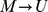 transition in that there are only two possible pathways: direct and GroELS-mediated. We construct an analytical model of GroEL-mediated folding in supplemental file Text S1. The performance of the GroELS chaperone system depends on both the capacity of the system (quantified by the concentration of total GroEL and total GroES), and the demands on the system (quantified by the concentrations at the entry points to the GroEL system – the unfolded and misfolded states). We study the percentage of GroELS-mediated folding trajectories as a function of the system capacity over the set of four proteins, each of which have different system demands.

**Figure 7:**
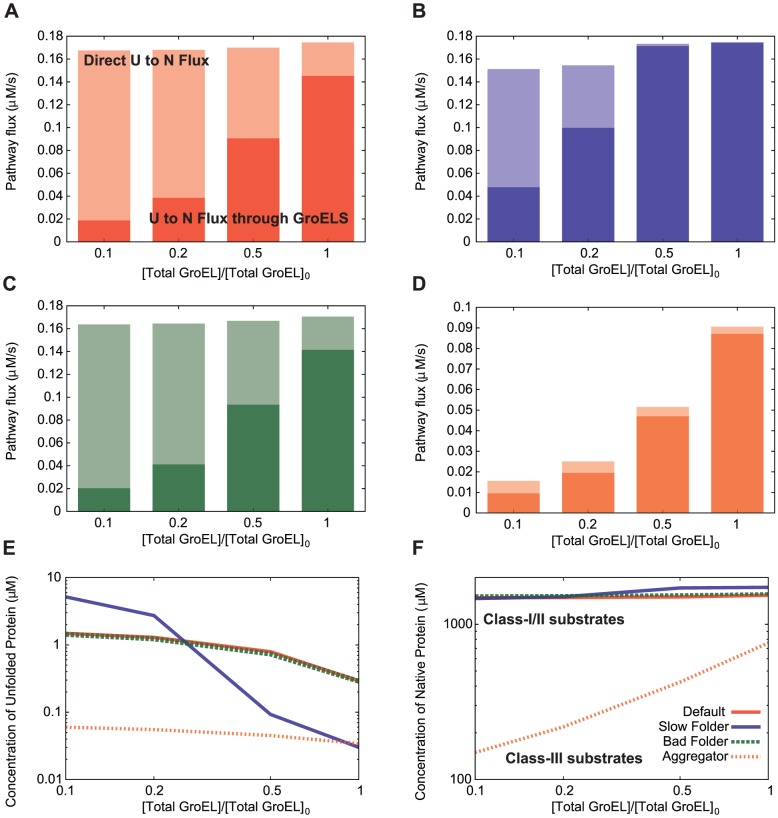
GroELS-mediated protein folding. (**a–d**) The pathway flux through GroELS (solid bars) is shown as well as the direct 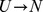 folding flux (transparent bars), for different concentrations of GroEL chaperone. The concentration of total GroEL is varied by a multiplicative factor ranging from 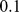 to 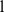. 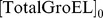, the reference value of GroEL chaperone, is equal to 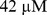, which is the value used in Section “Correcting misfolded states”. The concentration of GroES is varied along with GroEL by the same multiplicative factor, where 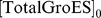 equals 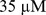. Panels (**a–d**) show data for the Default, Slow Folder, Bad Folder and Aggregator proteins respectively, with the colors corresponding to the legend of panel (**f**) (**e**) The concentration of unfolded protein at the evaluation time (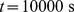). Colors for each protein correspond to those used in the above panels, and also to the legend in panel (**f**). (**f**) Concentration of the native state for each protein. Since the Default, Slow Folder and Bad Folder proteins do not strongly depend on the GroELS system, we call them “class-I/II substrates” according to the nomenclature of Kerner et al. [7]. In contrast, the concentration of the native state for the Aggregator protein strongly depends on the concentration of total GroEL, indicating that it should be called a “class-III” substrate.

Figures 7a–d show the pathway flux through the GroELS system (solid bars) as well as the direct flux (transparent bars) from the unfolded state to the folded state. The total concentrations of GroEL and GroES are varied together by a multiplicative factor ranging from 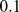 to 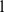 (plotted on the horizontal axes), where 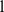 results in the concentrations used in the previous section. For all four of the proteins, both the absolute and percentage usage of the GroEL-mediated folding pathway goes down with decreasing total GroEL concentration. For three of the proteins (Default, Slow Folder, and Bad Folder, shown in panels a, b and c, respectively), this decrease is mostly compensated for by an increase in usage of the direct pathway. Of these, Slow Folder has the largest decrease in total folding flux, resulting in a decrease in native yield of 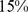, while Default and Bad Folder have decreases in native yield of 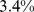 and 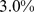, respectively. The compensation for lack of GroEL folding flux occurs by accumulation of unfolded protein that would otherwise enter the GroELS system (as the direct folding flux is given by 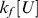) (Figure 7e). Therefore, GroEL is not needed for folding, but these proteins will take the GroEL pathway (almost exclusively) if it is available. As the compensation for the lack of GroELS occurs by building up population in the unfolded state, we note that without the GroELS system these proteins will be more vulnerable to degradation, and we expect to see a stronger dependence of native protein yield on GroELS at higher concentrations of the protease Lon.

In contrast, the folding flux for the Aggregator protein is highly dependent on the available concentration of GroEL. This is because the GroEL system acts as a “holder” to keep proteins from aggregating, and the extra unfolded protein resulting from its removal does not accumulate, but is transferred to the misfolded state, and subsequently aggregates. The Aggregator protein can thus be seen as a “class-III substrate” in that the total folding flux (and thus the concentration of the native state) is dependent on the availability of GroEL [7]. The other three proteins (Slow Folder, Default and Bad Folder) can be seen as “class-I” or “class-II substrates” of the GroEL system (see Text S1), in that they do not strictly require the GroELS system to fold. It is striking that the Slow Folder protein is not a “class-III substrate”, even though it was parameterized to be an optimal substrate of the GroELS system. We note that this is done using parameters at a lower synthesis rate. At this higher synthesis rate, the GroELS system primarily serves to rescue proteins from aggregation, as opposed to degradation, and as such the optimal clients of the GroELS system misfold and aggregate easily [21]. The biophysical profiles of top GroELS substrates in the presence of KJE at this synthesis rate are similar to that of the Aggregator protein (see Figure S4B of Powers et al. [21]).

It is important to note that the simulations conducted here only take into account one client protein at a time, whereas *in vivo*, there are about 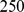 different proteins that act as GroELS substrates, which compete to bind to a shared pool of GroELS chaperones [7]. It is then easy to see how competition can arise between class-I/II and class-III substrates, as strong-binding class-I/II substrates would lower the effective concentration of total GroEL, reducing the yield of class-III substrates without increasing their yield of native protein. There should thus be an evolutionary drive to increase the binding affinity of class-III substrates in comparison to class-I/II substrates. In Text S1 we examine whether increasing the GroEL binding affinity for the Aggregator protein can compensate for lower concentrations of GroEL chaperone, and we find that it cannot. This underscores the importance of increasing binding affinity from the perspective of inter-protein competition.

## Discussion

We have used transition path analysis in combination with the FoldEco program to study the proteostasis network in *E. Coli*. The analysis reveals features of the network dynamics that are undetectable by observing concentrations of network components alone. For the misfolded to unfolded transition, we find that the usage of the KJE vs B+KJE systems depends mostly on the relative concentrations of the misfolded and aggregated states. We also observe that the efficiency, and hence the pathway probability, of the GroELS cycle depends mostly on folding kinetics of a client protein within the GroELS cycle. If the folding kinetics within the GroEL-GroES chaperone complex are the same as in bulk, in order for the GroELS system to increase native yields, either the degradation or aggregation processes need to be competitive with folding. We have also shown that modest adjustments in the binding affinities to the two chaperones DnaK and GroEL can control which chaperone system is used to correct misfolded states at a given synthesis rate.

This study serves as a proof of principle that a transition path analysis can be applied to proteostasis-type networks with little complication. We expect that this analysis will become more valuable as networks become larger and more interconnected, since the behavior of the transition paths will become less intuitive. The computational cost of the analysis is dominated by multiplications of matrices that are approximately size 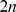 by 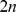, where 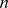 is the number of states in the network. Although matrix multiplication scales as 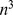, GPU architectures allow fast multiplications of large matrices (a two-GPU cluster can multiply matrices at a speed of 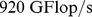 [24], hence matrices of size 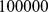 by 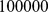 can be multiplied on a two-GPU cluster in about 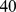 minutes). This would make the analysis presented here feasible on networks up to about 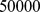 states with current hardware. For larger networks, rather than multiply matrices it would be easier to generate a large number of “psuedo-trajectories” using the state-to-state transition probabilities, and calculate mediation probabilities directly from the trajectories, as done in our previous work [23].

Mediation probabilities would be extremely challenging to measure experimentally, since they would rely on the tracking of single molecules *in vivo*. For instance, to determine the relative fraction of GroELS-, KJE- and B+KJE-mediated misfolded to unfolded transitions, one would need to distinguish between misfolded, unfolded, GroELS-bound, DnaK-bound and aggregated states in real time. However, even without verifying the mediation probabilities directly, the overall proteostasis network model can be verified by comparing the concentrations of species in the model with those from experiment over a range of system parameters (as is done for firefly luciferase in Powers et al. [21]). This prescribes a complex synergy between theory and experiment, where experiment is first used to parametrize the reactions, theory is used to construct a network, experiment then used again to validate the network, and theory used again for the network analysis described here.

## Methods

### Background: Proteostasis in *E. coli*

There are four main chaperone systems acting to maintain proteostasis in *E. coli*. The first is the Hsp70-like system, consisting of chaperones DnaK, DnaJ and GrpE (the KJE system). DnaK has a hydrophobic pocket that preferentially binds to unfolded peptides with exposed stretches of 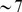 hydrophobic residues [9], [12]. Bound peptides can be locked in through the motion of a helical lid domain that is closed by the hydrolysis of bound ATP, which is regulated by the binding of co-chaperone DnaJ. After DnaJ unbinds, the binding of GrpE catalyzes ADP release, and subsequent ATP rebinding results in lid opening and release of the peptide. Because the protein is kept unfolded throughout the cycle, the KJE system can allow misfolded, aggregation-prone proteins to return to an unfolded state. A large part of the *E. coli* proteome (at least 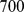 proteins) binds to DnaK [9], making the KJE system extremely important in preventing aggregation [4], [25].

The second is the Hsp60-like GroEL/GroES chaperonin system (GroELS), which is the only chaperone system that is absolutely necessary for the viability of an *E. coli* cell [26]. GroEL exists as two stacked seven-membered rings which form a cylindrical complex that is capable of encompassing a single protein, acting as an infinite-dilution cage. GroES forms a single seven-membered ring that acts as a cap to the cylinder, enclosing the protein. It has been shown that enclosure within the GroEL∶GroES complex can increase folding rates [27], although the chaperonin system works to prevent aggregation even when folding kinetics are unchanged. The unbinding of the GroES cap is mediated by allosteric ATP binding, and occurs after 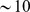 seconds [4], which gives the peptide time to fold in a sterically-confined environment that is isolated from other misfolded copies of the peptide that encourage aggregation. Discharged protein that is not folded can be rapidly rebound, and consequently many proteins are known to undergo many GroELS cycles before folding [27]–[29]. GroEL binds to a wide variety of proteins, comprising at least 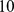 to 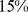 of cytosolic proteins under normal growth conditions [6]. An *in vitro* study by Kerner et al. shows that of about 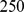 proteins that interact with GroEL, about 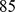 are absolutely dependent on the chaperonin system to fold [7]. However, a more recent study has shown that only 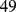 of these are strictly dependent (or “obligate”) on GroELS *in vivo* [8].

The KJE system can also cooperate with the 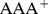 Hsp104-like chaperone ClpB to pull monomers from amorphous aggregates (the B+KJE system) [11], [13]–[15], [25]. ClpB is an oligomeric, ring-like machine that uses the energy from ATP hydrolysis to exert mechanical force on protein aggregates. Both DnaK and DnaJ are used to prepare aggregates for ClpB which then can extract monomers from the aggregate [30]. Two mechanisms have been proposed for the disaggregation mechanism of ClpB: one in which ClpB acts as a “crowbar” to break apart an aggregate [13], the other in which ClpB threads a single monomer through a central pore [14].

The last chaperone is trigger factor, which can bind to translating polypeptides and protect them from aggregation [7], [19]. As trigger factor only acts as a holder chaperone, trigger-factor–bound states cannot act as intermediates on transition paths between the major client protein states (e.g. native, unfolded, misfolded). We thus exclude trigger factor from our transition path analysis, and focus on the first three chaperone systems mentioned above.

### The FoldEco model

The FoldEco program was recently introduced to study the proteostasis of a client protein. It describes, in a holistic fashion, the synthesis, folding, misfolding, aggregation, degradation and recovery of misfolded and aggregated proteins through the KJE, GroELS and B+KJE chaperone systems. It uses coupled kinetic equations that evolve a particular set of initial conditions (which are the concentrations of each species in the system) forward in time, using reactions that are parameterized from *in vitro* experimental data.

In Figure 1 we show the network of client protein states used here. The nodes in the network are particular configurations of a single protein molecule, and mostly describe the formation and destruction of complexes with different chaperones in the proteostasis network. This can be compared with Figure 1 of Powers et al [21], where there is more information about the nature of the transitions, but does not explicitly include all of the connections between client states. We note that FoldEco also describes reactions that do not involve the client protein, such as the binding and unbinding of ATP from DnaK. We omit these from Figure 1 since they are not part of the network of client states. To simplify our analysis, we also connect the processes of degradation and re-synthesis through a “null” state. This does not affect our results, and allows us to examine the steady-state dynamics of a single protein traversing the network.

The rate constants for the transitions between the states in this network are determined from a large body of experimental literature. In theory these rate constants can be tailored in a protein-specific fashion to more accurately connect with experiment, although for simplicity we fix all but six rate constants, and the values for these fixed constants are given in Table S4 of Powers et al [21]. The same table also describes the initial concentrations of the chaperone species used here, which are reproduced in Table 2.

Although FoldEco is a powerful tool for synthesizing experimental data, there are some simplifications used by the model that affect our analysis. Firstly, it does not account for the effect of bacterial growth, which would lead to the dilution of proteins as they are being synthesized. One effect of this is that steady-states reached by FoldEco tend to have much larger concentrations of protein than are observed in experiment. Thus, in our analysis we do not analyze the networks at steady-state, we instead choose a common analysis time for each system (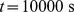). FoldEco also does not take into account the presence of the background proteome, and does not describe competition for binding to chaperones. Above, we study this competition indirectly by lowering the concentration of GroEL and GroES that is accessible to the client protein. We note that both of these limitations are planned to be addressed in future versions of FoldEco [21].

### Extracting a rate matrix

The kinetic equations in FoldEco are formulated as a set of equations that describe the time evolution of the concentrations of different client and non-client species in the system. For our analysis, we wish to convert this into a master equation of the form 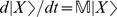, where 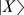 is a vector of the concentrations of different states in the model, and 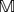 is a time-independent rate matrix, the elements 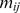 of which describe the rate of transition from state 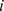 to state 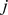. This allows for the transitions of a single tagged protein molecule to be tracked from state to state, and for the analysis of its dynamical properties. The first complication to arise is that some of the states in the network involve multiple copies of the client protein. For instance, a GroEL-GroES complex can accommodate two client proteins, one in the *cis* ring and one in the *trans* ring. It is important to maintain a distinction between proteins in the same complex if they are in different states (e.g., one is folded and the other is unfolded). We thus artificially separate the multi-client complex states into two states, depending on which protein is the tagged protein. This allows us to track the tagged protein in a continuous fashion once the complex has dissolved.

Aggregated structures are also multi-client states, but to rigorously keep track of a specific monomer in a large aggregate would be unfeasible: describing aggregates up to 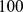 monomers in length would require over 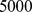 states for each aggregate-containing species in the network. This is because a monomer of size 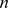 would require 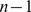 distinct states that distinguish the position of the monomer within the aggregate. Furthermore, it is not clear whether or not the order in which monomers are added to the aggregate affects the order in which they would be removed by ClpB. We thus assume, upon removal of a monomer from an aggregate of size 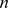, that the probability of the tagged protein being removed is equal to 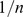. The assumption of the indistinguishability of monomers is reasonable for amorphous aggregates, and would also be reasonable for highly ordered aggregates if ClpB could act via a “crowbar”-type mechanism [13], allowing monomers to be extracted from the middle of a structure. We note that for beta amyloid, both amorphous (preamyloid) and fibrillar aggregates can be observed *in vitro*, depending on the conditions [31].

After a proper set of states is established, a second complication arises in building the master equation, as some of the differential equations are nonlinear (e.g., the rate of aggregation of two monomers depends on the square of the monomer concentration), and many of the rates depend on the concentrations of non-client protein species, which are changing over time. We instead cast the problem as 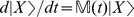, where the elements of 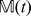 depend on the average concentrations of the network components, and thus on the time 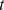. In principle, at long times the concentrations of the different species in the model will reach steady state, and the elements of 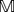 will be time-independent, but this limit does not always exist, especially for conditions in which runaway aggregation occurs. In order to employ the same protocol for a broad range of simulation conditions, we instead choose an analysis time of interest (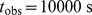) and use the rate matrix calculated at that time for analysis. We then assume that the elements of this matrix are approximately constant on the timescale of the transition paths. This assumption is addressed in Text S1.

### Getting mediation probabilities

Once we have obtained the rate matrix as calculated at the observation time, we use a method similar to that proposed in our previous work [22] to obtain mediation probabilities. Previously, modified rate matrices were diagonalized in order to determine infinite-time behavior. As we have found some of the matrices here to be unstable to diagonalization, we instead use a slightly modified approach. Firstly, the rate matrix is converted into a transition probability matrix, in which the elements are equal to the probabilities of transition between states in a given amount of time, 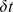. To find an appropriate 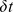 we find the fastest rate in the system (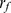), and then set 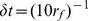. The equation 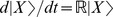, yields, for small 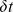


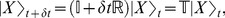
(1)where 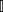 is the identity matrix, and 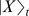 denotes the value of the vector 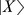 at time 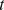. The nondiagonal elements of the transition matrix 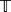 are then equal to 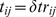, and the diagonal ones to 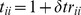.

This transition matrix is then modified in the same manner as in our previous work [22]. Here we describe the method in brief, focusing on the parts that differ from our previous implementation. Consider a modified transition probability matrix with “sinks” at two states 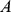 and 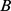, created by setting the nondiagonal elements in columns 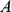 and 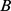 uniformly to zero, and setting the diagonal elements to 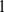. The long-time dynamics using this new matrix, 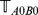, reveals committor probabilities for each state 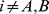 as follows:
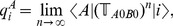
(2)where 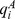 is the probability of reaching 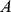 before 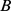, given starting in state 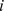, and 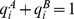.

In order to determine mediation probabilities, we calculate “conditional committors”, such as 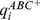, which is the probability, when starting in 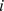, of reaching 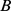 before 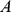, having gone through a third state, 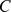. The probability of reaching 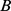 before 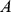 having *not* gone through 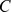 is denoted 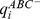. Conservation of probability now gives the equality 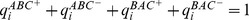. These conditional committors are computed using an extended probability matrix, where two ensembles of states are used: one in which 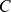 has been visited so far along the trajectory, the other in which 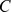 has not been visited. Sinks are put into the matrix at 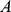 and 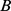, in both ensembles, and conditional committors are determined using the elements of the infinite-time extended probability matrix, in a manner similar to Equation 2. In practice, 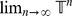 is computed by iteratively squaring the matrix 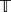 until the probability in the non-sink states is less than 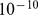. The number of iterations required is less than or equal to 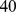 for all matrices used here.

## Supporting Information

Figure S1 **Concentrations of protein species.** The concentration of native, unfolded, misfolded and aggregated species for each of the four characteristic proteins at all of the synthesis rates examined in Figure 2. The concentration of aggregated protein shown here was calculated by counting the total number of monomers present in aggregates of every size.
(EPS)

Figure S2
**Concentrations of DnaJ-containing species for the Slow Folder protein at low binding factors.** The concentration of free dimeric DnaJ (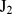), and dimeric DnaJ bound in a ternary complex with unfolded protein and ATP-bound DnaK (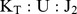) or ADP-bound DnaK (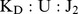) over the range of binding factor (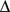) examined in Figure 6b. As 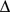 decreases, the rate constant for binding of DnaK to unfolded or misfolded protein increases. For low 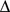, this causes an increase in the concentration of the ternary unfolded protein-DnaJ-DnaK complexes, which reduces the concentration of free DnaJ dimers, which in turn decreases the efficiency of the KJE cycle.(EPS)

Figure S3
**Checking the constant rate matrix assumption.** The absolute value of the difference between pathway probabilities computed using transition probability matrices evaluated at 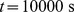 and 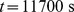 is shown. The largest difference between the pathway probabilities is less than 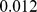 for the KJE pathway at a ribosome activation rate of 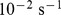.(EPS)

Figure S4
**Error check at steady state.** A comparison of weights calculated from the rate matrix with weights obtained by normalizing the steady state concentration for the Default protein at a synthesis rate of 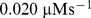. The line 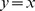 is shown for comparison. Agreement starts to break down for weights less than 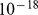 due to computational rounding errors (not shown).(EPS)

Figure S5
**GroEL-mediated folding as a function of free ATP-bound GroEL.** The ratio of GroEL-mediated flux (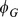) over direct folding flux (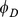) is plotted against the concentration of free ATP-bound GroEL, for the GroELS mediation data in Section “GroELS-mediated folding” (four proteins at four concentrations of total GroEL). The data for each protein is shown as points, and the fit to 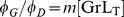 is shown. The fitting parameter 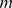 is seen as the 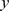-intercept (or the height of the line) on the log-log plot.(EPS)

Figure S6
**Native protein yields as a function of GroEL binding affinity.** The yield of native protein in 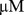 is shown for a broad range of relative GroEL binding affinities. The latter is a multiplicative factor that adjusts the binding rates of unfolded and misfolded protein to GroEL complexes. Curves are shown for the four different total concentrations of GroEL/GroES used in Figure 7.(EPS)

Text S1 **Section I: Verifying the constant rate matrix assumption.** Section II: Error check at steady state. Section III: An analytical description of the percentage of GroELS-mediated folding. Section IV: Classifying the proteins using the scheme of Kerner et al. Section V: Examining the effect of altering GroEL binding affinity.
(PDF)
