# Supplementary material for: Quantifying Chaperone-Mediated Transitions in the Proteostasis Network of E. coli
Source: PLoS Comput Biol. 2013 Nov 14;9(11):e1003324. doi: 10.1371/journal.pcbi.1003324 (PMC3828153; doi:10.1371/journal.pcbi.1003324)
Supplement: Text S1 — Section I: Verifying the constant rate matrix assumption. Section II: Error check at steady state. Section III: An analytical description of the percentage of GroELS-mediated folding. Section IV: Classifying the proteins using the scheme of Kerner et al. Section V: Examining the effect of altering GroEL binding affinity. (PDF) [file pcbi.1003324.s007.pdf]

Quantifying chaperone-mediated transitions in the proteostasis network of *E. coli* Supporting Information

Alex Dickson, Charles L. Brooks III

I. VERIFYING THE CONSTANT RATE MATRIX ASSUMPTION

To address the constant rate matrix assumption, we first determine the time duration of the transition paths studied here. The slowest transition paths that we find are for the Aggregator protein (see Section *Four characteristic protein profiles* for the protein definitions) at the highest synthesis rate studied here (0.175 μM/s). When computing the mediation probabilities for this matrix, we find that 99% of the probability in non-trap states has been transferred to trap states within 19 iterations, which corresponds to a transition time of 1700 s. We then compare pathway probabilities obtained with the matrices at $t = 10000$ s and $t = 11700$ s, and find that they agree to within 0.012 for each pathway (Figure S3). This justifies our assumption that the transition matrices are roughly constant on the timescale of the transition paths we consider here.

II. ERROR CHECK AT STEADY STATE

To demonstrate that the rate-matrix approach is implemented correctly, we pick a particular protein (Default at a ribosome activation rate of 0.001 s^{-1}), and run it until it reaches steady state, which in this case was 2×10^7 s. We then compare these converged concentrations with weights computed from the rate matrix as follows. The rate matrix is converted to a transition probability matrix, which is multiplied by itself (i.e. squared) until it converges. Each column of the resultant matrix is then identical to the steady-state weights of all the species in the system. The first column is chosen, and the weights of the aggregated species are then divided by the number of monomers in the aggregate. This is to convert from a "monomer concentration" to a species concentration. To further motivate this requirement: consider two aggregates with equal concentration in solution, one with 2 members and the other with 10. A monomer is 5 times as likely to be in the 10-mer than the 2-mer, and thus the 10-mer will have a weight (or a "monomer concentration") that is 5 times that of the 2-mer. A comparison of the weights computed from the rate matrix, and those computed from normalized steady state concentrations is given in Figure S4. The agreement is excellent, which validates our implementation.

III. ANALYTICAL DESCRIPTION OF PERCENTAGE GroELS FOLDING

Due to the relative simplicity of the U $\rightarrow$ N folding transition, we can derive an analytical expression for the percentage of GroELS mediated trajectories. The direct folding flux is given by $\phi_D = [U]k_f$, where $[U]$ is the concentration of unfolded protein and k_f is the folding rate. The GroELS system is accessible from two states: U and M. Each of these species can bind to either ATP-bound GroEL ($\mathrm{GrL_T}$), or GroEL with no nucleotide bound (GrL). We denote the binding rates of U to $\mathrm{GrL_T}$ and GrL as k_{UT} and k_{UG}, respectively, and label the corresponding rates from the misfolded state as k_{MT} and k_{MG}. We can then define a committor probability ($p_{X,S}$), for each GroELS-bound state S, as the probability of reaching the native state before returning to the prior, non-GroELS entry state X (which is either U or M). The folding flux through the GroELS system is then

$$\phi_G = [U][GrL]k_{UG}p_{U,GrL:U} + [U][GrL_T]k_{UT}p_{U,GrL_T:U} \quad (1)$$

$$+ [M][GrL]k_{MG}p_{M,GrL:M} + [M][GrL_T]k_{MG}p_{M,GrL_T:M}. \quad (2)$$

Using the assumption that $[GrL_T] >> [GrL]$, we have

$$\frac{\phi_G}{\phi_D} = \frac{[GrL_T]}{k_f}\left(k_{UT}p_{U,GrL_T:U} + \frac{[M]}{[U]}k_{MT}p_{M,GrL_T:M}\right), \quad (3)$$

where on the right hand side the only variables that change as a function of total GroEL are $[\mathrm{GrL_T}]$ and $[M]/[U]$.

Using the data for GroELS mediation for the four proteins at four different concentrations of total GroEL, we show that plots of $\frac{\phi_G}{\phi_D}$ vs $[\mathrm{GrL_T}]$ can be fit to the function $f(x) = mx$, where m is a parameter unique to each protein (Figure S5). This shows that the concentration of free $\mathrm{GrL_T}$ is key to understanding the changes in the GroEL

mediation in the bar graphs of Figure 7, and that $[M]/[U]$ is relatively constant for each protein over the range of total GroEL examined. The fitting parameter m is equal to $0.11, 0.38, 0.56$ and 0.64 $\mu\mathrm{M}^{-1}$ for Slow Folder, Default, Aggregator and Bad Folder respectively, and involves the demand on the GroELS cycle ($[U]k_{UT} + [M]k_{MT}$), the committor functions from each entry point, and the folding rate. We note that if characteristic values of the fitting parameter for GroELS client proteins can be determined, then this linear dependence could be exploited. GroELS mediation probabilities could then be accessed experimentally using the concentrations of free GroEL at a range of total GroEL concentrations. This is significant as it connects a dynamic quantity (the ratio of two pathway fluxes) to the concentration of a single species in the proteostasis network.

IV. CLASSIFYING PROTEINS USING THE SCHEME OF KERNER ET AL

In Kerner et al. [1], proteins are labeled either "class-I", "class-II" or "class-III" based on their observed folding behavior both with and in the absense of chaperones. Class-I proteins can recover a large portion of their enzymatic activity (e.g. 55% for enolase) at 37 C, 0.5 μM with no chaperones. They can then reach 100% of enzymatic activity upon the addition of GroEL. In contrast, class-II proteins show no enzymatic activity at 37 C with no chaperones, though activity is restored by GroEL, and partially restored by DnaK; some activity is seen with no chaperones at 25 C.

To distinguish between class-I and class-II in our simulations we turn protein production off by setting the concentration of ribosome to zero, start with 0.5 μM of unfolded protein, and run forward in time without chaperone until the concentrations stop changing. Doing this we obtain native yields of 10%, 46%, 10% and 5% for Default, Slow Folder, Bad Folder and Aggregator, respectively. We thus classify Slow Folder as a class-I substrate due to its modest native yield in the absence of chaperone, and the others as either class-II/III. Based on the analysis in the main text (see Figure 7), we conclude that Default and Bad Folder are class-II, and Aggregator is class-III. The relevant difference between the Slow Folder and the others is its relatively strong bias against the misfolded state ($K_m = 0.1$, in contrast to 40, 100 and 200 for Aggregator, Default and Bad Folder, respectively). This is intuitive, as class-I proteins should have smooth energy landscapes that are guided toward the folded state, with a very high energy barrier to aggregation.

V. EXAMINING THE EFFECT OF GroEL BINDING AFFINITY

Figure 7 shows that as the concentration of GroEL and GroES decreases, the folding flux along the GroELS pathway decreases, which for Aggregator results in a significant decrease in native yield. Here we examine to what extent this effect can be mitigated by an increase in GroEL binding affinity. The affinity is adjusted by increasing the binding rates for U + GrL, U + $\mathrm{GrL_T}$, M + GrL, and M + $\mathrm{GrL_T}$, while keeping the unbinding rates constant. We also increase the rates of either U or M binding to the trans ring of a preformed $\mathrm{GrL_D}$:X:GrS complex, where X is either N, U or M. These rates are adjusted together by a multiplicative factor, which we refer to as the relative GroEL binding affinity. In Figure S6 we show the yield of native protein for the Aggregator as a function of binding affinity for the different concentrations of total GroEL studied in Figure 7 in the main text. Figure S6 shows that changes in total GroEL concentration are much more significant than increases in GroEL binding affinity: a reduction of GroEL concentration by half (such as going from $[\mathrm{GroEL}]/[\mathrm{GroEL}]_0 = 1.0$ to $[\mathrm{GroEL}]/[\mathrm{GroEL}]_0 = 0.5$) cannot be compensated for even by an increase in binding affinity by four orders of magnitude.

We emphasize that this does not incorporate the effects of competition of multiple proteins for a shared pool of GroEL. In that situation, an increase in binding affinity for a given protein would be coupled to an increase in the *effective* concentration of GroEL that is accessible to that protein species, which, as is shown here, would be very effective in increasing native yields of that protein.

[1] M. J. Kerner, D. J. Naylor, Y. Ishihama, T. Maier, H.-C. Chang, A. P. Stines, C. Georgopoulos, D. Frishman, M. Hayer-Hartl, M. Mann, et al., Cell **122**, 209 (2005).
